# Germline *SMARCA4* mutations in patients with ovarian small cell carcinoma of hypercalcemic type

**DOI:** 10.1186/s13023-015-0247-4

**Published:** 2015-03-15

**Authors:** Joanna Moes-Sosnowska, Lukasz Szafron, Dorota Nowakowska, Agnieszka Dansonka-Mieszkowska, Agnieszka Budzilowska, Bozena Konopka, Joanna Plisiecka-Halasa, Agnieszka Podgorska, Iwona K Rzepecka, Jolanta Kupryjanczyk

**Affiliations:** Department of Pathology and Laboratory Diagnostics, Maria Sklodowska-Curie Memorial Cancer Center and Institute of Oncology, Roentgena 5, 02-781 Warsaw, Poland; Department of Genetics, Maria Sklodowska-Curie Memorial Cancer Center and Institute of Oncology, Warsaw, Poland

**Keywords:** Small-cell carcinoma of hypercalcemic type, Immature teratoma, Parotid gland carcinoma, *SMARCA4* mutation, Germline mutation, Ovarian cancer

## Abstract

**Background:**

*SMARCA4* mutations have recently been identified as driving lesions of the ovarian small cell carcinoma of hypercalcemic type (SCCHT). Familial occurrence of this neoplasm was described previously.

**Methods:**

We looked for germline *SMARCA4* alterations in eight patients with the SCCHT. DNA was extracted from probands’ and their relatives’ blood. The *SMARCA4* coding sequence, previously found altered in all the tumors, was PCR amplified and sequenced in the germline DNA.

**Results:**

Two patients carried a heterozygous germline *SMARCA4* alteration: c.3760G > T and c.2352insG, respectively. The analysis of the probands’ next of kins revealed that the c.3760G > T mutation was inherited by the proband and her sister from their father, and the sisters’ four children also carried the mutation. The proband’s sister was diagnosed with a carcinoma of the parotid gland at age 2. A brother of the other proband was tested negative.

**Conclusions:**

Our study suggests that some women develop the ovarian SCCHT due to the inherited or possibly *de novo*-occurring germline alterations in the *SMARCA4* gene, however, its penetrance appears limited. Nevertheless, because of high aggressiveness of the SCCHT, a molecular diagnostics of the *SMARCA4* gene and careful follow-up should be offered to patients with this cancer and their families.

## Background

A small cell carcinoma of the ovary of hypercalcemic type (SCCHT) is a rare and highly malignant neoplasm affecting young females. In the largest published group of patients the age of onset ranged from 9 to 43 years (median 24 years). Microscopically, the predominant population is that of small undifferentiated ovoid cells. There are also foci of large cells with eosinophilic cytoplasm, with or without eccentrically displaced nuclei (rhabdoid features). The architecture is heterogeneous and disordered, with pseudofollicles [1,2].

The SCCHT grows rapidly and shows poor response to available chemotherapies. Overall survival of affected patients is generally short; it largely depends on a clinical stage and ranges from 5 months to several years at the FIGO I stage, and 2–23 months at the FIGO III stage [3].

Familial occurrence of the SCCHT has been described in a few reports, and it led to death of young first degree relatives [4-7]. Thus, identification of patients at risk of development of this neoplasm and its early detection might improve the prognosis.

Until recently, the SCCHT remained a mystery in terms of histogenesis and molecular background [2,8-10]. In year 2013, our research group has described its development in association with an ovarian immature teratoma and brought to light its similarity to atypical teratoid/rhabdoid tumor of the central nervous system (AT/RT). Based on these findings and the fact that some AT/RTs may develop due to *SMARCA4* gene mutations [11], we performed an analysis of this gene and identified *SMARCA4*-inactivating mutations in two SCCHT analyzed [12]. Subsequently, other groups have also found *SMARCA4* mutations in this neoplasm [13-15] and all these findings suggest that the SCCHT is an ovarian rhabdoid tumor.

The *SMARCA4* gene encodes an ATP-dependent helicase BRG1 which belongs to the SWI/SNF (mating type SWItching defective/Sucrose Non Fermenting) complex and is involved in epigenetic regulation of gene expression via chromatin remodeling. The importance of different *SMARCA4* alterations, their penetrance and traits of inheritance are just beginning to be explored. In this study we present germline alterations of the *SMARCA4* gene in two patients diagnosed with the SCCHT; one of these alterations was present in three generations of the proband’s relatives.

## Methods

### Patients and tumors

Initially, we identified eight SCCHT with *SMARCA4* mutations; normal tissue from all the patients was available. Two of the eight patients had germline *SMARCA4* alterations and they are subjects of this study (the analysis of somatic molecular changes in the larger group including the eight tumors is a matter of a separate study, Dansonka-Mieszkowska et al. unpublished). In the next step, normal tissue used for DNA extraction was obtained from the relatives of both probands. All adult subjects gave written informed consent and the study was approved by the local ethics committee (ref. no 13/2008).

#### Proband 1 (PJK1182)

A 35 y.o. patient underwent cesarean section in the 33rd week of pregnancy during which the SCCHT of the right ovary was removed. Within this tumor there was a focus of immature teratoma [12]. Subsequent staging laparotomy revealed International Federation of Gynecologists and Obstetricians (FIGO) stage IIIB neoplasm. The patient received 3 lines of combination chemotherapy and died 12 months after the initial diagnosis due to progression of the disease.

#### Proband 2 (tumor 392)

**Figure 1:**
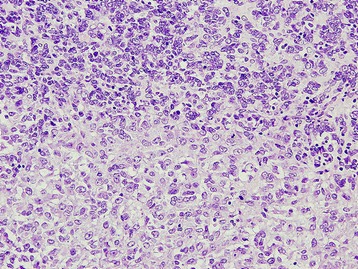
**Small cell carcinoma of hypercalcemic type (proband 2, tumor 392).** Histological pattern (HE staining, 200x).

A 21 y.o. patient was admitted to the hospital because of a tumor of the left ovary, ascites and right-sided hydrothorax. She underwent radical gynecological surgery and was diagnosed with FIGO IV ovarian SCCHT (Figure 1). The patient received 6 courses of paclitaxel and carboplatin and died 6 months later due to progression of the disease.

### Analysis of *SMARCA4* gene mutations

Genomic DNA was isolated from formalin-fixed paraffin-embedded or frozen tissues using the QIAamp DNA Extraction Kit (Qiagen, Hilden, Germany), according to the manufacturer’s instructions. The *SMARCA4* coding sequence [GenBank: NG_011556.1], previously found altered in the tumors, was PCR amplified and sequenced in the germline DNA. The PCR and sequencing primer sequences of exon 16 (Table 1) were taken from the publication by Medina et al. [16]; primer sequences for exon 26b (Table 1) were designed personally using the Primer3 algorithm. The PCR mixture was prepared according to the protocol provided with AmpliTaq Gold PCR kit (Life Technologies, Foster City, USA). Thermal cycling conditions for both exons were as follows: an initial denaturation at 95°C for 5 min; followed by 36 cycles of sample denaturation at 94°C for 30 s, primers annealing at 59°C for 30 s, and product extension at 72°C for 30 s, followed by a final 7 min extension at 72°C. All DNA samples were sequenced in the ABI PRISM 3100 sequencer with the use of BigDye Terminator Cycle Sequencing Kit (v.3.1) (both from Life Technologies), according to the manufacturer’s recommendations.

**Table 1:** **The PCR and sequencing primers for the exons in which mutations were detected**

**Exon**	**Forward primer**	**Reverse primer**
16*	AGGACCCTCTGGTGTCCGAC	TGTTGCTGGCAGCGGGTAC
26b	CTCAACGTGGACCAGAAGGT	TCAGCCCACACTCCCTTTAC

*Primers based on published sequences [16].

## Results

### Mutations in the *SMARCA4* gene and the family history of cancer

#### Proband 1

**Figure 2:**
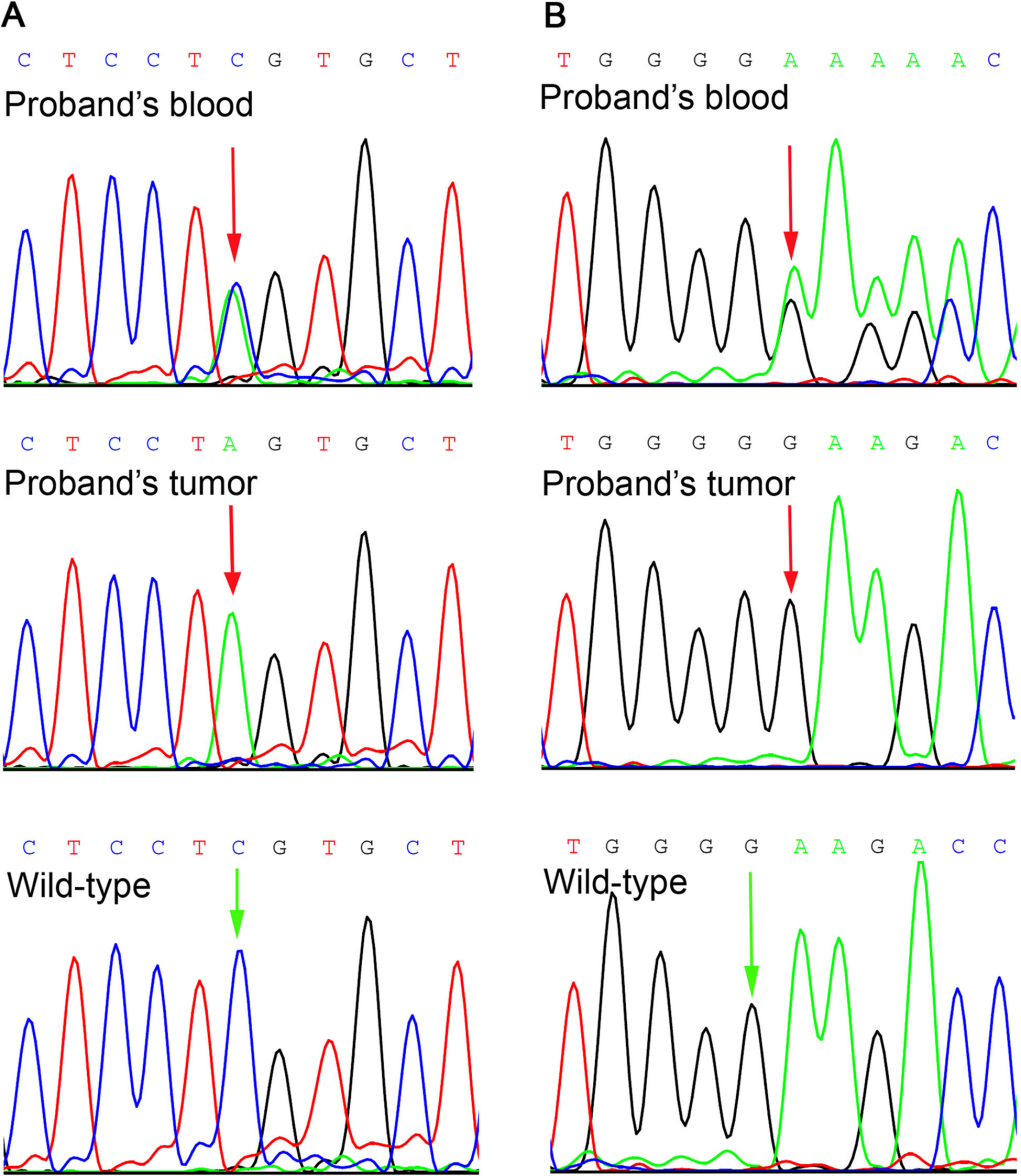
**Chromatograms of germline** ***SMARCA4*** **mutations. (A)** c.3760G > T in proband 1. **(B)** c.2352insG in proband 2.

**Figure 3:**
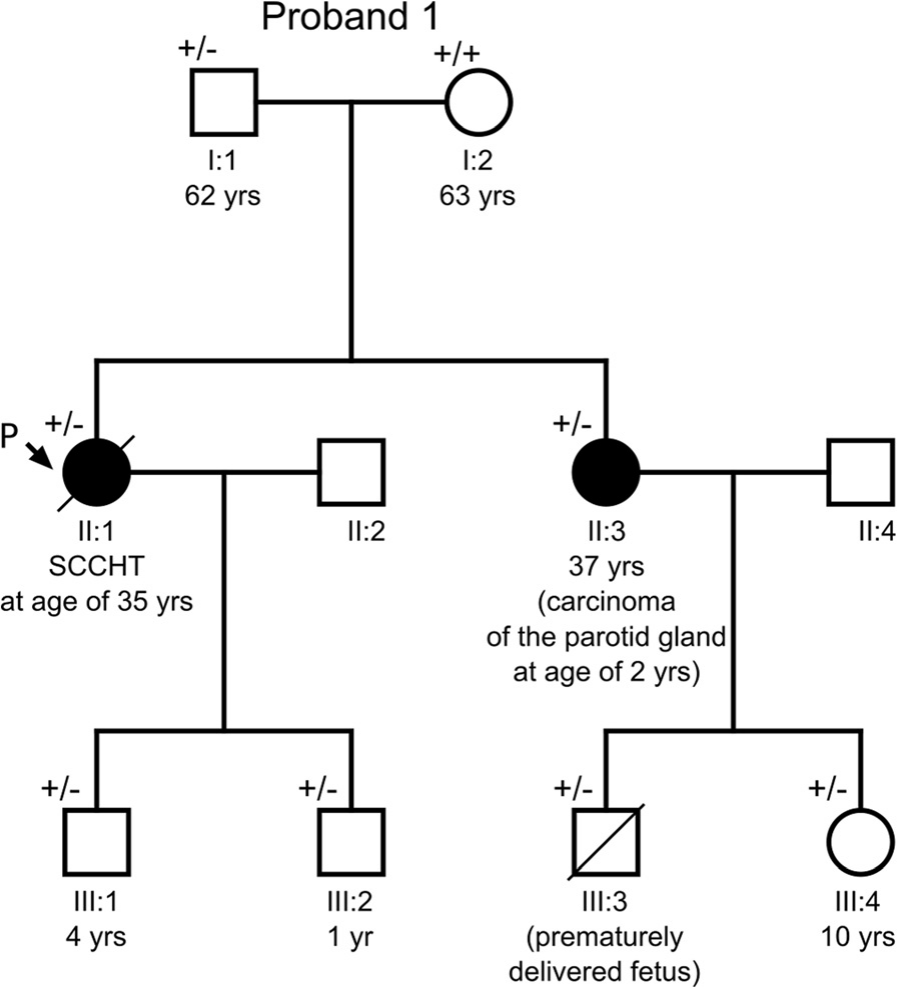
**A pedigree of the proband’s 1 family.** The proband (P, II:1) with SCCHT had a germline mutation in *SMARCA4* exon 26 (c.3760G > T) inherited from her father (I:1). This mutation was also identified in the proband’s two sons (III:1, III:2), sister (II:3) and in the sister’s two children (III:3, III:4). (+/−) heterozygous mutation carrier in the germline; (+/+) wild-type in the germline. A diagonal line through a symbol indicates that the person is deceased.

A *SMARCA4* nonsense mutation c.3760G > T with the loss of heterozygosity was found in the ovarian SCCHT from this patient. This alteration led to premature termination of the BRG1 protein, p.(Glu1254*) (see Table 2). Analysis of germline DNA from the proband revealed the same mutation in one allele (Figure 2A). A subsequent analysis of her family has shown the same mutation in the germline of her six first- and second-degree relatives (Figure 3).

**Table 2:** ***SMARCA4*** **mutations found in two patients with ovarian small cell carcinoma of hypercalcemic type**

	**Age**	**FIGO stage**	**SMARCA4 expression** ^**a**^	***SMARCA4*** **mutation**	
				**Tumor DNA**	**Germline DNA**
**Proband 1**	35	IIIB	Negative	Exon 26 c.3760G > T; p.(Glu1254*) (homozygous)	Heterozygous
**Proband 2**	21	IV	Negative	Exon 16 c.2352insG; p.(Lys785Glufs*39) (homozygous)	Heterozygous

^a^SMARCA4 expression evaluated with Brg-1 clone G-7antibody [12]. *means a stop codon.

The only sibling (sister) of the proband was diagnosed with carcinoma of the parotid gland at age 2. The carcinoma was described as solid basal, partially cylindromatous with extensive cord-like infiltration and hyalinisation, difficult to interpret, presumably adenoid cystic carcinoma (the tumor had been evaluated by several expert pathologists, slides and blocks no longer available). After surgery she received radiotherapy (4600 R/g) and has been remaining free of cancer for 35 years now. No other neoplasms in the first and second-degree relatives of the proband were reported (the father and his two sisters and one brother).

#### Proband 2

**Figure 4:**
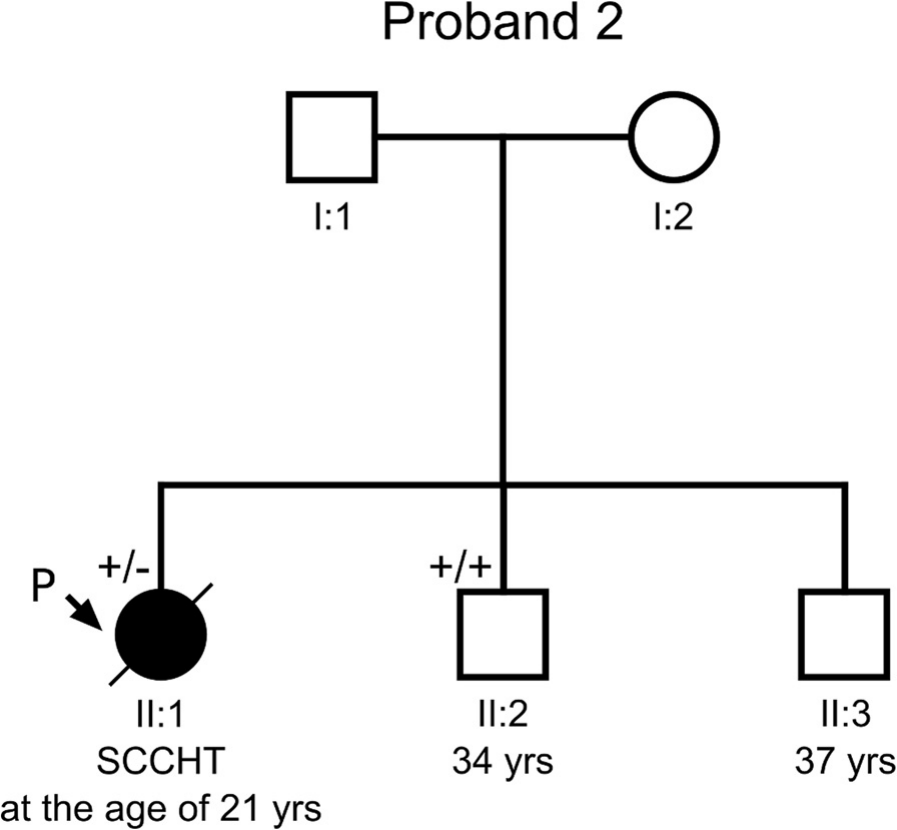
**A pedigree of the proband’s 2 family.** The proband (P, II:1) with SCCHT had a germline mutation in *SMARCA4* exon 16 (c.2352insG), while her brother (II:2) turned out to be unaffected. The proband’s parents (I:1, I:2) and her second brother (II:3) refused to attend the study. (+/−) heterozygous mutation carrier in the germline; (+/+) wild-type in the germline. A diagonal line through a symbol indicates that the person is deceased.

Another *SMARCA4* homozygous mutation – insertion of a single guanine nucleotide c.2352insG (Figure 2B), was found in the other tumor investigated. This frameshift alteration resulted in a premature termination of the BRG1 protein p.(Lys785Glufs*39) (Table 2). The same mutation, yet heterozygous, was identified in the proband’s blood. Among the proband’s family members (parents, two brothers), only one brother agreed for examination of his germline DNA and was tested negative (Figure 4). According to the information obtained from this brother, there was no history of cancer in their parents and the parents’ siblings (altogether 6).

## Discussion

*SMARCA4* gene alterations have recently been found to underlie a development of the ovarian small cell carcinoma of hypercalcemic type [12-15]. In this study we present two patients with novel germline *SMARCA4* alterations. The pedigree chart of one family shows that the c.3760G > T; p.(Glu1254*) *SMARCA4* mutation, occurring across three generations, was inherited in an autosomal manner.

This is only the fifth family with the ovarian SCCHT and the *SMARCA4* germline mutation, after four pedigrees published by Witkowski et al. [15]. In each family of the latter study, the mutation of *SMARCA4* gene was carried by a mother and a daughter, and all of them were affected by an ovarian cancer, mostly the SCCHT. In one of those families, similarly to our results, the mutation was also identified in the proband’s father. Interestingly, in both our and the Witkowski’s et al. [15] study, all examined offsprings carried the germline *SMARCA4* mutation detected in their ancestors. Since not all mutation carriers have been diagnosed with cancer, the penetrance of the *SMARCA4* gene appears incomplete. This has also been raised by Hasselblatt et al. [17] on the basis of data on children with AT/RT carrying a germline *SMARCA4* mutation, and their families.

Recently, other groups have also found germline *SMARCA4* mutations in patients with the SCCHT [13,14], however, they did not present a pedigree analysis. In accordance with our results, all *SMARCA4* germline mutations described to date caused a premature stop codon or altered splice site, and were associated with somatic loss of the wild-type allele and SMARCA4/BRG1 protein expression in the tumors [11,13-15,18,19].

In mammalian cells, the SWI/SNF complex exhibits the tumor suppressor activity, and SMARCA4/BRG1 protein is one of its two most essential subunits. Malfunction of the complex may negatively affect cell migration, nuclear hormone receptors signaling, embryonic stem cell programs, lineage-specific differentiation and cell proliferation [20]. The both germline alterations in the *SMARCA4* gene reported by our research team occurred in the important ATPase domain (functioning as the motor units that convert ATP energy to mechanical movement) [21].

Mutations in the *SMARCA4* gene appear to be associated with various cancers including malignant melanoma, non-small cell lung cancer, head and neck and pancreatic cancer [22]. Families with germline *SMARCA4* mutations have been reported to develop malignant rhabdoid tumors (SCCHT, AT/RT, renal rhabdoid tumor) and yolk sac tumor (YST), all belonging to the group of embryonal tumors [15,17,18]. Our study apparently adds two tumor types to the spectrum of cancers observed in carriers of *SMARCA4* mutations, i.e., the ovarian immature teratoma and carcinoma of the parotid gland. The immature teratoma showed diminished expression of the BRG1 protein compared with normal tissues, thus it presumably retained one wt *SMARCA4* allele [12]. As to the parotid gland carcinoma, we do not know whether there was LOH at the gene locus in this tumor. However, other authors describing *SMARCA4* mutations in different cancers did not evaluate LOH as well (Shain & Pollack [22]). Haploinsufficiency of the *SMARCA4* gene should also be taken into account as a potential mechanism of tumor progression, since it was previously reported by Bultman et al. for heterozygous *Smarca4* (+/−) mice developing mammary tumors [23].

Previously, we demonstrated foci of immature teratoma (a germ cell tumor) in two ovarian SCCHT (including the first one presented in this study) [12]. Thus, some SCCHT appear to originate from immature teratoma, as many other secondary tumors developing in this pluripotential neoplasm, and this does not contradict their assignment to the group of rhabdoid tumors. In one of their recent studies, Witkowski et al. [19] found a germline *SMARCA4* mutation in a patient with an original diagnosis of ovarian immature teratoma and suggested that it might have been a malignant rhabdoid tumor. As we have demonstrated, rhabdoid tumors may develop in association with the immature teratoma and this neoplasm may be observed in the *SMARCA4* mutation carriers.

The penetrance of the *SMARCA4* gene appears to be limited, and factors that modify it are to be discovered. Based on pedigrees described by Witkowski et al. [15] and our study, it appears that female carriers are more susceptible to cancers than males. Due to high aggressiveness of the SCCHT, it seems reasonable to offer the patients with this cancer and their families a molecular diagnostics of the *SMARCA4* gene. *SMARCA4 m*utation carriers should be subjected to thorough observation, including ultrasonographic and magnetic resonance imaging of potentially affected organs. Since the SCCHT develops to age 44, an option might be a prophylactic oophorectomy in older patients who completed their families, provided that they would be informed that the risk of the disease has not yet been determined. It appears that women from families with more than one female members affected by the SCCHT are at particular risk of developing this neoplasm.

## Conclusions

In conclusion, our study suggests that some women develop the ovarian SCCHT due to the inherited or possibly *de novo*-occurring germline alteration in one *SMARCA4* gene allele, with subsequent loss of the wild type allele. More families with *SMARCA4* mutations have to be described to precisely assess the risk of the SCCHT and other neoplasms, and to establish adequate medical care of the mutations carriers.
